# Association of Choline Intake with Blood Pressure and Effects of Its Microbiota-Dependent Metabolite Trimethylamine-N-Oxide on Hypertension

**DOI:** 10.1155/2022/9512401

**Published:** 2022-08-25

**Authors:** Guo-dong He, Xiao-cong Liu, An-shang Lu, Ying-qing Feng

**Affiliations:** ^1^Department of Cardiology, Guangdong Cardiovascular Institute, Guangdong Provincial People's Hospital, Guangdong Academy of Medical Sciences, Guangzhou 510080, China; ^2^Research Department of Medical Sciences, Guangdong Provincial People's Hospital, Guangdong Academy of Medical Sciences, Guangzhou 510080, China; ^3^Forevergen Biosciences Center, Guangzhou 51000, China

## Abstract

**Background:**

The association of total choline (TC) intake and its metabolite trimethylamine-N-oxide (TMAO) with hypertension and blood pressure (BP) has not been elucidated.

**Methods:**

For the population study, the association of TC intake with hypertension, as well as blood pressure, was determined through logistic along with multiple linear regression analysis from the National Health and Nutrition Examination Survey 2007 to 2018, respectively. For the animal experimental study, spontaneously hypertensive rats (SHRs) were assigned to the water group or water containing 333 mg/L or 1 g/L TMAO group. After 22 weeks treatment of TMAO, blood pressure measurement, echocardiography, and histopathology of the heart and arteries were evaluated.

**Results:**

No significant association of TC with hypertension was observed but the trend for ORs of hypertension was decreased with the increased level of TC. Negative association between TC and BP was significant in quintile 4 and quintile 5 range of TC, and the negative trend was significant. The SHR-TMAO groups showed significant higher urine output levels in contrast with the SHR-water group. No difference of diastolic BP was observed, but there was a trend towards lower systolic BP with the increase doses of TMAO in the SHR group. The SHR 1 g/L TMAO rats had a remarkably lower systolic blood pressure than the SHR-water group. Echocardiography showed a diastolic dysfunction alleviating effect in the 1 g/L TMAO group.

**Conclusion:**

High TC intake was not linked to elevated risk of hypertension. An inverse relationship of choline intake with systolic BP was observed. The mechanism for the beneficial effect of TC might be associated with the diuretic effect of its metabolite TMAO.

## 1. Introduction

Hypertension is the most frequent cardiovascular risk factor and represents the leading modifiable risk factor for death resulting from atherosclerosis and cardiovascular disease [1]. Epidemiological study showed that unhealthy dietary and lifestyles contributes to abnormal regulation of blood pressure (BP) [2]. Elevated BP may result in vascular wall remodeling including endothelial dysfunction and vascular stiffness [3]. All of these changes in vascular result in the development of atherosclerosis and cause tissue injuries in the renal and cardiovascular systems [4].

Choline, a nutrient for humans, has remarkably extensive range of biological roles in human health, as well as disease [5]. However, the diverse sources of choline such as fish, red meat, eggs, and whole grain have been postulated to positively and negatively impact cardiovascular diseases (CVD) [6]. The excessive and unabsorbed choline would be metabolized to yield trimethylamine (TMA) during gut dysbiosis which would be transported to liver through portal circulation then further oxidized to trimethylamine-N-oxide (TMAO) by hepatic flavin monooxygenases [7]. Numerous epidemiological reports documented links elevated TMAO concentrations to CVD [8–10]. However, systematic review and meta-analysis of prospective studies revealed that choline was not associated with CVD [11]. Two studies of the association between choline and CVD mortality showed inconsistent findings [12, 13]. Besides, choline-deficient diets could disrupt the intestinal barrier associated with nonalcoholic steatohepatitis which would result in hyperglycemia and obesity [14–16]. Therefore, whether CVD is linked to choline intake of remains unknown. Besides promoting atherosclerosis in mice, TMAO was also documented to prolong and aggravate the hypertensive influences of angiotensin II in rats [17, 18]. Although it suggested a potential impact of this choline originated metabolite on BP, the recent cross-sectional study does not reveal a positive link between choline intake with BP but an inverse connection with hypertension in women [19]. Another population-based cohort study revealed that high dietary intake of choline was associated with the low risk of developing hypertension [20]. Nevertheless, the associations of choline intake with hypertension and BP among older adults were dependent on other risk factors, such as body mass index (BMI) and comorbidity status [21]. Therefore, the relationship of choline intake with BP is still obscure, and the mechanism of choline regulating BP remains elusive.

Herein, we evaluated the potential relationship of total choline (TC) intake with hypertension and BP through the data from the 2007 to 2018 cross-sectional United States National Health and Nutrition Examination Survey (NHANES). To better comprehend the effect of TMAO, the metabolite of choline, on blood pressure and its complications, we investigated the chronic 22-weeks-long treatments with different doses of TMAO in spontaneously hypertensive rats (SHRs).

## 2. Material and Methods

### 2.1. Study Population

**Figure 1:**
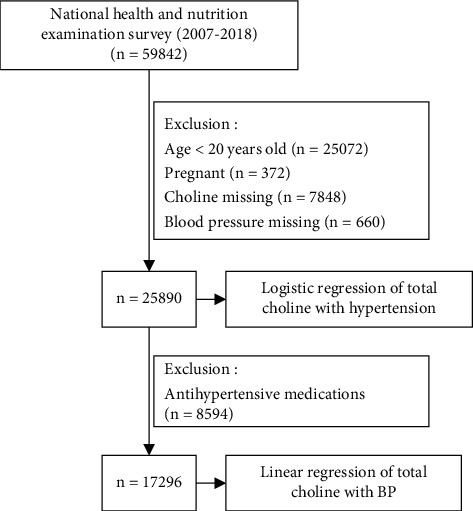
Study cohort.

We used data from 2007 to 2018 from the NHANES (http://www.cdc.gov/nchs/nhanes/), a continuous, cross-sectional study of the noninstitutionalized, civilian US population, with data released in two-year cycles. NHANES is designed to reflect the U.S. civilian noninstitutionalized population via a complex, multistage probability sample [22]. All NHANES protocols were approved by the National Center for Health Statistics Research Ethics Review Board, and all participants gave written informed consent [22]. The analytic sample consisted of nonpregnant individuals aged ≥ 20 years with completed 24 h dietary recalls, and participants who had missing choline data and BP were omitted (*n* = 25890; Figure 1).

To analyze the choline-hypertension status association, we considered the following: (1) doctors' diagnosis reports ≥ 2 times of hypertension diagnosis, (2) reported via antihypertensive medication(s), and (3) had mean systolic blood pressure (SBP) ≥ 140 mmHg or diastolic blood pressure (DBP) ≥ 90 mmHg to be hypertensive (*n* = 11032) while nonhypertensive individuals (*n* = 14858) were defined as participants not meeting the above three criteria.

For the analysis of the association between choline and BP, we omitted participants under antihypertensive prescriptions (*n* = 8594), yielding 17296 study participants.

### 2.2. Measurement of Choline Intake

Trained interviewers conducted two 24-hour dietary recalls by using validated automated multiple-pass approach. According to nutrient values of the Food and Nutrient Database for Dietary Studies, total dietary choline was evaluated [23]. Label information was employed to compute the supplemental choline. The sum of supplemental and dietary choline resulted in overall choline intake for every recall day. The average overall choline intakes across the two recall days served as the continuous variables in the analyses.

### 2.3. Baseline Variables Collection

During the household interviews, the baseline variables consisting of age, gender, ethnicity, marital status, ratio of family income to poverty, smoking status, and level of education were acquired from the participants. Weight along with height measurements was acquired to determine BMI (kg/m^2^) by standardized methods [22]. The estimated glomerular filtration rate (eGFR) was determined via the Chronic Kidney Disease Epidemiology Collaboration equation (mL/min/1.732 m^2^) [24]. Intakes of total calories, folate, vitamins B6 and B12, protein and fat, cholesterol, and sodium from foods, as well as supplements, were determined from the average of two 24 h recalls, with computations as documented above for choline.

### 2.4. Animals Experimental Protocol

Animal care and experimental procedures were performed under the guidelines of the Research Ethics Committee of Guangdong Provincial People's Hospital.

Male SHRs and normotensive Wistar-Kyoto (WKY) rats were provided by Beijing Vital River Laboratory Animal Technology Co. Ltd. The rats were kept in the Experimental Animal Center of Forervegen (Guangzhou, China) and were housed in groups of three animals in polypropylene cages on a 12-hour light/12-hour dark cycle with temperature at 22–23°C, humidity at 45–55%, and standard laboratory diet and water ad libitum. The 333 mg/L and 1 g/L of TMAO were selected in order to increase the plasma TMAO by 3-6 times and 9-18 times, respectively, to mimic possibly physiological low-dose and high-dose TMAO concentrations based on previous study [25, 26]. Seven- to eight-week-old SHRs (*n* = 18) were randomly assigned to water group (drinking tap water) or water containing 333 mg/L or 1 g/L TMAO group (TMAO, product number: T1362, Tokyo Chemical Industry, Japan). WKY (*n* = 6) rats served as the normotensive controls for discriminating between hypertension-dependent and age-dependent histopathological alterations in SHRs.

### 2.5. Effect of Gut Microbial-Related Choline Metabolite TMAO on the Onset and Progress of Hypertension and Its Complications in 30-Week-Old Rats

After 22-week treatment with water or TMAO, systolic along with diastolic blood pressure of the rats was monitored using a tail blood pressure measuring instrument (Zhenghua Biologic Apparatus Facilities, Anhui, China). To evaluate the 24-hour water and food balance, rats were kept in metabolism cages for 2 days. For echocardiography, we anesthetized 30-week-old rats with 1.5–2% isoflurane and then kept warm them on a heated platform. During echo, we monitored the vital signs. To assess the cardiac functions, the Vevo 2100 (Visual Sonics, Canada) with a linear probe at 21 MHz was employed to monitor the echocardiographic parameters. Volumes and functional parameters were measured and analyzed by a blinded researcher. After echocardiographic recordings, the plasma, hearts, and arteries were harvested for further analysis. Rat frozen tissue samples were sliced at 4 *μ*m intervals and fixing (in 4% PFA) performed. Masson staining of the sections (with the Masson Trichrome Staining Kit) was done as described by the manufacturer (Absin, Shanghai, China).

### 2.6. Plasma TMA and TMAO Evaluation

A 50 ± 5 *μ*L plasma sample was placed in a 2 mL centrifuge tube after being thawed on ice. 10 *μ*L isotopic internal standard solution and 0.45 mL methanol were added and then mixed by vortex. After being centrifuged under 14000 g, the supernatant was then injected for analysis. Plasma TMA along with TMAO were assessed using an Agilent 1290 Infinity Ultra Performance Liquid Chromatograph system on a HILIC column (Waters, BEH HILIC 2.5 *μ*m, 2.1 mm by 100 mm column) coupled with an AB SCIEX 5500 QTRAP triple-quadrupole mass spectrometer. Eluent A was acetonitrile, and Eluent B was water consisting of 10 mM ammonium formate buffer (pH 3.5). The gradient elution program was as follows: 0 min = 90% B, 1.5 min = 90% B, 4.5 min = 87% B, 7 min = 85% B, 7.5 min = 50%B, 10 min = 50% B, 10.5 min = 90% B, and 14 min = 90% B. Before injecting the next sample, the column was equilibrated with the initial mobile phase for 5 min. The flow rate was constant at 0.4 mL/min, and the column temperature was set at 25°C. The mass spectrometer was performed in a positive switch mode. The ion transitions were *m*/*z* 76.1 > 58.0 for TMAO, *m*/*z* 60.1 > 44.1 for TMA, *m*/*z* 69.1 > 49.1 for TMA-D9, and *m*/*z* 85.1 > 66.0 for TMAO-D9. The ESI source conditions consisted of source temperature of 550°C, ion source gas 1 of 55, ion source gas 2 of 55, curtain gas of 40, and ion sapary voltage floating of 4500 V. The multiple-reaction monitoring method was used for mass spectrometry quantitative data acquisition. The calibration curve ranges were 1–5000 ng/mL for TMAO and TMA.

### 2.7. Statistical Analysis

TC level was divided into five groups according to quintiles. Baseline continuous and categorical variables were reported as mean ± standard deviation (SD) or percentages. The linear trend for baseline characteristics was tested by linear or logistic regression whenever appropriate. Logistic regression and multiple linear regression models were utilized to understand the association of choline-hypertension status and choline-BP, respectively. The model I was unadjusted. The model II was based on specific covariates: age, gender, and race; model III includes BMI, ratio of family income to poverty, marital status, education, eGFR, activity, smoke, energy, protein, fat, sodium, cholesterol, folate, vitamins B-6, vitamins B-12, and comorbidities (hypercholesterolemia and diabetes). We imputed missing values of covariates (1-8%) using cohort-specific mean values. To investigate if the association varied by age at baseline (<60 or ≥60 years), gender (female/male), smoking (yes/no), BMI (<25 or ≥25 kg/m^2^), and eGFR (<90 or ≥90 mL/min/1.732 m^2^), we performed subgroup analyses.

In the animal experiments, the differences in the mean values between groups were analyzed using Student's *t*-test or one-way analysis of variance (ANOVA), followed by Bonferroni's test for multiple comparisons. The Shapiro-Wilk test was applied to test normality of the distribution. Results are given as *mean* *values* ± *SD*.

*p* < 0.05 (2 sided) was considered significant. The statistical analyses were all implemented in R V.4.0.3 (R Foundation for Statistical Computing).

## 3. Results

### 3.1. Baseline Characteristics

The demographic characteristics of the study participants on the basis of the five TC levels are indicated in Table 1. The average age of the 25890 adults was 50.42 years, with approximately half them being women (51.6%). Non-White race was 56.9%, whereas white race was 43.1%. About three-quarters of the participants self-identified as high school or above. Fifty percent of the participants were married. 65.6% of the sample were not current smokers. Male, smoking, and moderate-intensity activity participants were more likely to be with higher TC values. The odds of hypertension in this analytic sample were 42.6%.

### 3.2. Relationship of TC with Hypertension

Table 2 illustrates the adjusted ORs (95% CIs) of hypertension in the five groups of TC levels. No positive association of TC levels with hypertension was found. However, ORs of hypertension were remarkably negatively linked to TC levels in the upper range (Q3-Q5) in the unadjusted model I (*p* for trend < 0.001), especially in the Q5 range (OR: 0.81, 95% CI: 0.75, 0.87, *p* < 0.001). In model II, when the ORs were adjusted for age, race, and gender, only the Q3 range of TC level was negatively correlated with hypertension (OR: 0.90, 95% CI: 0.82, 0.99, *p* < 0.033). Although assessment with multivariable adjustments (model III) exhibited no remarkable association of TC levels with hypertension, the trend for ORs of hypertension was decreased with the increased level of TC (*p* = 0.041).

### 3.3. Relationship of TC with BP

For analysis of TC-BP association, we omitted the utilization of antihypertensive medications (*n* = 8594). The multivariable adjustments of linear regression showed the negative relationship of TC intake with SBP (Table 3) and SBP and little to no relationship of TC with DBP was reported (Table 4). In model I, TC intake was positively linked to BP could be found. When the model of TC-BP association was adjusted for age, gender, and race (model II), TC intake was positively linked to DBP could still be found in Q4 and Q5 range of TC (*β*: 0.575, SE: 0.28, *p* = 0.040; *β*: 0.62, SE: 0.287, *p* = 0.031, respectively) while TC was remarkably inversely correlated with SBP. In model III, when adjusted for all covariates, little to no relationship of TC with DBP was reported any more. However, negative TC-SBP association was still significant in the Q4 and Q5 range of TC (*β*: -1.082, SE: 0.451, *p* = 0.017; *β*: -1.408, SE: 0.619, *p* = 0.023, respectively), and the negative trend was increased with the increased level of TC (*p* = 0.013). Scatter plots of systolic pressure and diastolic pressure versus choline intake were shown in Figure S1a and S1b (Supplementary Figure S1).

We conducted subgroup analyses to explore whether the effects of TC differed with age, gender, BMI, eGFR, and with or without smoking and tested for interactions (Table 5). There was significant interaction with age, BMI, and eGFR in the relationship of TC level with BP (*p* for interaction < 0.001, 0.015, and <0.001, respectively). The association of total choline intake with SBP of hypertension differed by age and BMI. Higher total choline intake tended to be associated with lower SBP in those aged ≥ 60 years but not in the aged < 60 years. Besides, the association was stronger in the participants whose BMI < 25 kg/m^2^.

### 3.4. Effect of Gut Microbial-Related Choline Metabolite TMAO on the Development of Hypertension

**Figure 2:**
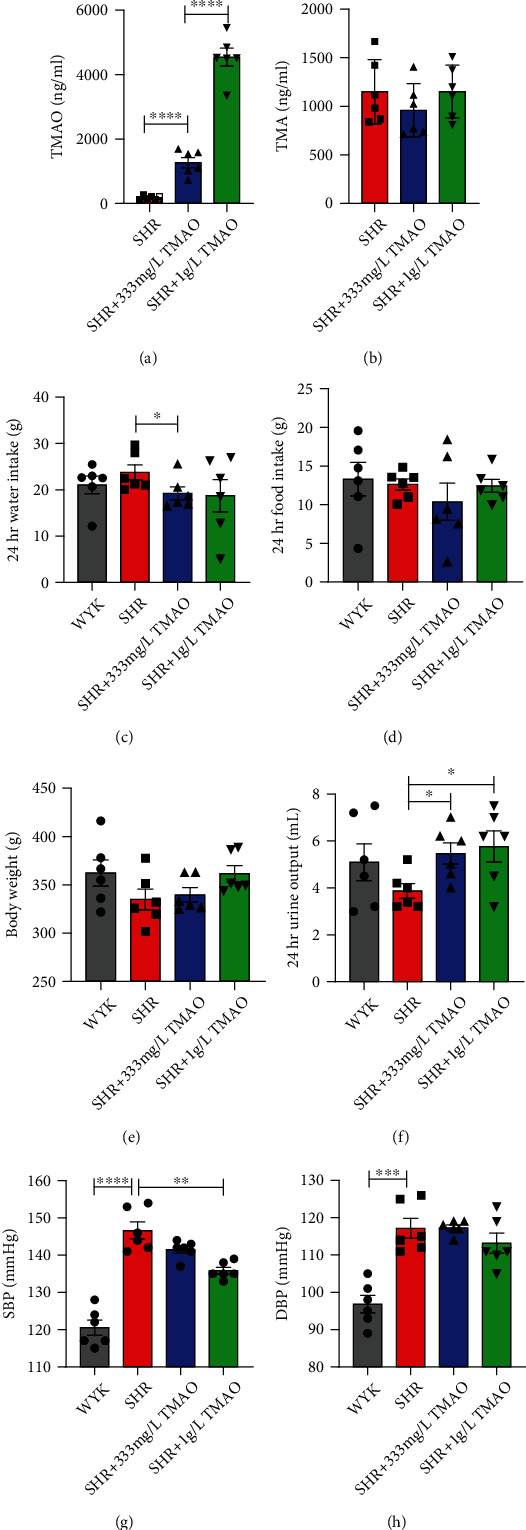
Effect of gut microbial-related choline metabolite TMAO on the development of hypertension. After 22 weeks treatment with water or TMAO, variation in the plasma (a) TMAO concentration, (b) TMA, (c) 24 hours water intake, (d) food intake, (e) body weight, (f) 24-hour urine output, and (g) systolic and (h) diastolic blood pressure of rats in each group. Results are mean ± SD. ^∗^*p* < 0.05; ^∗∗^*p* < 0.01; ^∗∗∗^*p* < 0.0005; ^∗∗∗∗^*p* < 0.0001. Grey bars represent Wistar-Kyoto (WKY) rats (*n* = 6); red bars represent spontaneously hypertensive rats (SHRs) treated with water (*n* = 6); blue bars and green bars represent SHRs treated with water containing 333 mg/L (*n* = 6) and 1 g/L TMAO (*n* = 6), respectively.

The TMAO plasma level of rats in the SHR 333 mg/L TMAO group was significantly higher than the SHR-water group (6-fold increase, p < 0.0001) (Figure 2(a)). The SHR 1 g/L TMAO group showed a 3-fold increase of plasma TMAO level compared with the SHR 333 mg/L group. And there was no significant difference in plasma TMA levels among groups (Figure 2(b)).

Rats in the SHR 333 mg/L TMAO group showed moderately lower water intake than SHR-water rats (*p* = 0.0411, Figure 2(c)). There was no significant difference in food intake and body weight between groups (Figures 2(d) and 2(e)). However, the SHR 333 mg/L and 1 g/L TMAO groups showed a significant higher urine output level in contrast with the SHR-water group (*p* = 0.0166 and *p* = 0.0268, respectively, Figure 2(f)).

Although no difference of diastolic blood pressure was observed, there was a trend towards lower systolic blood pressure with the increase doses of TMAO (*p* = 0.1775), and the SHR 1 g/L TMAO rats had a remarkably lower systolic blood pressure than SHR-water group (*p* = 0.0013, Figures 2(g) and 2(h)).

### 3.5. Effect of Gut Microbial-Related Choline Metabolite TMAO on Hemodynamic and Cardiac Parameters

**Figure 3:**
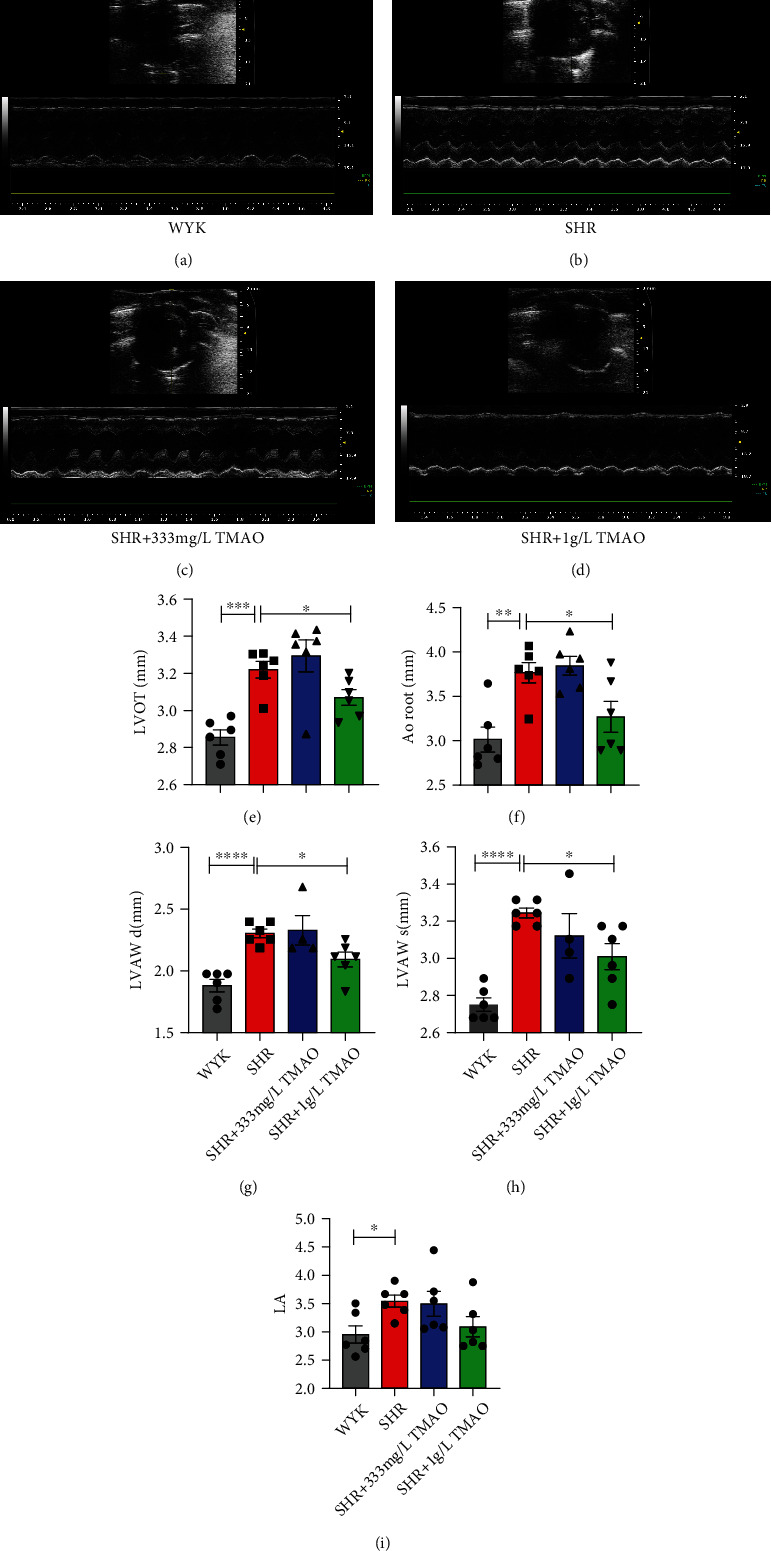
Effect of gut microbial-related choline metabolite TMAO on hemodynamic and cardiac parameters. The echocardiography pictures of 30-week-old WKY rats, SHR treated with water, and SHR treated with 333 mg/L and 1 g/L TMAO in drinking water for 22 weeks are shown in Figures 3(a)–3(d). Variation in (e) LVOT (left ventricular outflow tract), (f) AoRoot (aortic root), (g) LVAWd (left ventricle anterior wall thickness in diastole), (h) LVAWs (left ventricle anterior wall thickness in systole), and (i) left atrial dimension (LA) in each group. Results are mean ± SD. ^∗^*p* < 0.05; ^∗∗^*p* < 0.01; ^∗∗∗^*p* < 0.0005; ^∗∗∗∗^*p* < 0.0001. Grey bars represent Wistar-Kyoto (WKY) rats (*n* = 6); red bars represent spontaneously hypertensive rats (SHRs) treated with water (*n* = 6); blue bars and green bars represent SHRs treated with water containing 333 mg/L (*n* = 6) and 1 g/L TMAO (*n* = 6), respectively.

The echocardiography pictures of 30-week-old WKY rats, SHR treated with water, SHR treated with 333 mg/L and 1 g/L TMAO in drinking water for 22 weeks are shown in Figures 3(a)–3(d). There were no significant differences among groups in basic echocardiographic parameters, including heart rate, cardiac output, stroke volume, fractional shortening, and ejection fraction. And no significant differences were observed between SHR-water and SHR 333 mg/L rats in echocardiographic parameters, including LVOT (left ventricular outflow tract), AoRoot (aortic root), LVAWd (left ventricle anterior wall thickness in diastole), and LVAWs (left ventricle anterior wall thickness in systole) (Figures 3(e)–3(g)). However, the SHR 1 g/L group showed significant decrease on LVOT, AoRoot, LVAWd, and LVAWs (*p* = 0.0365, *p* = 0.0368, *p* = 0.0119, and *p* = 0.0101, respectively, Figures 3(e)–3(g)). And the SHR 1 g/L group showed a trend towards a lower left atrial dimension (LA) level (*p* = 0.0563, Figure 3(h)).

### 3.6. Effect of Gut Microbial-Related Choline Metabolite TMAO on Histopathology of the Heart and Arteries

**Figure 4:**
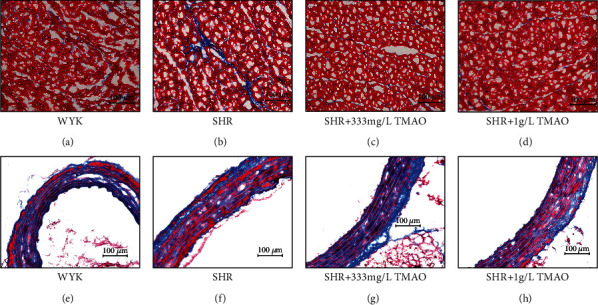
Effect of gut microbial-related choline metabolite TMAO on histopathology of the heart and arteries. Masson staining of the left ventricular tissue from the above (a) Wistar-Kyoto (WKY) rats, (b) spontaneously hypertensive rats (SHRs) treated with water, and SHRs treated with water containing (c) 333 mg/L and (d) 1 g/L TMAO. Masson staining of aorta from (e) WKY rats, (f) SHRs treated with water, and SHRs treated with water containing (g) 333 mg/L and (h) 1 g/L TMAO.

The left ventricular myocardial and aorta sections were colorized with Masson staining to visualize connective tissue (Figure 4). As judged visually, hyperplasia in the connective tissue in the myocardium was greater in the SHR-water group in contrast with other groups (Figures 4(a)–4(d)). However, no significant difference was visualized in histological sections of cardiomyocyte morphology and aorta between the groups (Figures 4(a)–4(h)).

## 4. Discussion

Here, we confirmed that higher TC intake was not linked to an elevated risk of hypertension via the large-scale data from the 2007 to 2018 cross-sectional NHANES. Moreover, we observed a trend for decreasing ORs of hypertension with the increased level of TC and found an inverse relationship of choline intake with SBP. Besides, our initial research showed that the prospective mechanism for the overall beneficial impact of choline may be associated with the diuretic effect of its microbiota-dependent metabolite TMAO.

Choline was an essential molecule for the synthesis of phosphatidylcholines [6]. Free choline would be reabsorbed in the small intestine [27], and only the excess levels are metabolized by the gut microflora to produce TMA in the colon [28, 29]. Only a small fraction of the microorganisms (less than 1%) was in the intestine harbor the genes required for TMA production, but they were sufficient to produce TMA [30]. TMA and TMAO levels were associated with the activity of the phylum Firmicutes and Proteobacteria [31]. Furthermore, TMA and TMAO levels were also associated with an elevated Firmicutes/Bacteroidetes ratio [32] as Bacteroidetes was not able to produce TMA [33]. Moreover, the differences in microbiota composition were due to differences in diet, lifestyle, and environment [34]. Therefore, the style of choline intake would impact its metabolism though the alteration on microbiota composition.

A few studies had reported choline intake could protect against CVD and no connection of choline intake with CVD mortality [11, 13, 35, 36]. Until recently, a prospective study reported that higher habitual dietary intake of phosphatidylcholine was associated with an increased risk of CVD mortality in the US population [12]. Nonetheless, there was a considerable controversy regarding the role of dietary choline intake because several studies had also reported that choline was associated with unfavorable cardiometabolic risk [8, 37–42]. Besides, there was a growing body of literature implicating choline metabolite TMAO in CVD risk [8, 10, 42, 43]. Therefore, it has been opined that higher choline consumption might promote the CVD process. Our study was primarily motivated by the question of whether TC intake is linked to hypertension and blood pressure in a large-scale study.

In this study, it was no surprise that our data do not support an adverse event of TC intake on BP because a recent study had documented a negative relationship of choline with SBP in adults aged ≥ 65 years, and higher TC intake appeared to be linked to lower odds of hypertension among women base on a relatively small-scale study [19]. Our study extended these previous findings by indicating that the higher TC intake inversely related to SBP. In this study, it was noteworthy that the highest quintiles' (Q5 and Q4) TC intake showed remarkably negative association with BP. However, the data of models for the BP outcome were not entirely congruent with the models for the hypertension outcome, in which only a trend for decreasing ORs of hypertension with the increased level of TC was observed. Besides, no association between choline and DBP was found in this study. Omitting participants with taking antihypertensive medications (33% loss of hypertensive cases) in the models for the BP outcome did not seem to account for these discrepancies because same results was achieved when participants with taking antihypertensive medications were not omitted (Supplementary Table S1). This inconsistency might arise from differences in the age, BMI, and eGFR according to the subgroup analyses in this study. It was of note that modifying factors could impact the associations of choline intake with BP levels and hypertension risk. A recent NHANES analysis in older adults reported that these associations might depend on other risk factors, especially for BMI [21]. In our study, the association of total choline intake with SBP was modified not only by BMI. Specifically, higher total choline intake tended to be associated with lower SBP in those aged ≥ 60 years but not in the aged < 60 years. Besides, the association was stronger in the participants whose BMI < 25 kg/m^2^.

Taken together, our study (indicating higher choline intake was not linked to hypertension but inversely related to BP) and other studies showing the connection of choline consumption with CVD incidence, we could hypothesize that excess choline which would be metabolized to produce TMAO may be beneficial on hypertension development. Therefore, we investigated the chronic 22-week-long treatments with different doses of TMAO in spontaneously hypertensive rats (SHRs) to explore the mechanism of the overall protective effect of dietary choline on BP/hypertension status. Our study provides evidence that a high level of TMAO did not exert an unfavorable effect but a beneficial effect which was associated with diuretic in SHRs.

TMAO was a metabolite from phosphatidylcholine and choline which produce by gut microbiota and hepatic flavin-containing monooxygenase 3 [44]. Moreover, fish intake which is considered beneficial for health provided a direct source of TMAO [45]. Interestingly, TMAO was upregulated in seafood thriving in the deep sea at high pressures which seems to protect the proteins from harm. However, increased plasma TMAO contents had been linked to major adverse CVD events and type 2 diabetes in diseased populations [46, 47] and had been used as a disease biomarker [48, 49]. As discrepancies were reported from different observational studies, the prospective function of TMAO in cardiometabolic diseases needs further investigation.

Previous study showed that moderate TMAO supplementation would increase plasma TMAO contents by four- to fivefold while exerting a beneficial effect in heart failure rats [25] and reducing the diastolic dysfunction in the pressure-overloaded hearts of hypertensive rats [26]. In this study, not only alleviating diastolic dysfunction but also a beneficial impact on systolic blood pressure in hypertensive rats was observed when plasma TMAO levels were increased by nine- to eighteenfold. However, there was no significantly unfavorable effect of this high plasma TMAO level in hearts and aorta. We also evaluated the effect of plasma TMAO levels by three- to sixfold, but the systolic and diastolic blood pressure showed no significant difference. However, it was noteworthy that the diuretic effect of plasma TMAO levels by three- to sixfold was almost the same as the higher plasma TMAO (increased by nine- to eighteenfold) which could prevent the pressure-overloaded hearts from fibrosis. Therefore, the mechanism of the higher plasma TMAO levels exerting a beneficial impact on systolic blood pressure in hypertensive rats might be not just the diuretic effect. It was still noteworthy that when animal subjects were Sprague-Dawley rats, TMAO was found to prolong the hypertension induced by ANG II infusion [17]. Thus, further studies might be required to fully elucidate the effect of TMAO on secondary hypertension.

Multiple limitations of the present study should be acknowledged. First, the 24 h recall is just a snapshot in time of an individual's diet. Therefore, the reported nonsignificant findings might be accounted for intraindividual variation and the lack of plasma choline measurement. Second, as this study did not adjust for complex survey design, the results might not be generalizable to the U.S. population. Besides, the long timeframe could also bias the estimates. Third, to prevent stress-related circulatory complications in SHRs, biochemical, hemodynamic, and histopathology measurements of rats were performed only at the end of the experiment. Fourth, the sources of choline may be essential to the connections with disease risk, and further investigations were required. Finally, the mechanism of the effect of gut microbial-related choline metabolite TMAO on hypertension required further study on not only the diuretic effect but also others, such as protein structure protective effect [50] and endoplasmic reticulum stress-alleviating effect [51].

In conclusion, higher TC intake was not linked to an elevated risk of hypertension. A trend for decreasing ORs of hypertension with the increased level of TC and a negative relationship of choline intake with SBP was observed in a large-scale cross-sectional study. The prospective mechanism for the overall beneficial effect of choline might be associated with the diuretic effect of its microbiota-dependent metabolite TMAO. Further studies were still required to investigate the effects and mechanism of choline and its metabolite TMAO on the circulatory system which might serve as a potential dietary strategy for preventing CVD development.

## Figures and Tables

**Table 1:** Baseline demographics and clinical characteristics according to total choline quintiles.

Variables	Total	Total choline intake (mg)					^a^ *p* for trend
		Q1 (0.15-196.9)	Q2 (196.9-262.95)	Q3 (263.0-332.8)	Q4 (332.85-434.25)	Q5 (434.3-2211.75)	
*n*	25890	5179	5179	5177	5177	5178	
Age, years	50.42 ± 17.54	50.91 ± 18.29	51.46 ± 18.12	51.06 ± 17.50	50.42 ± 17.16	48.25 ± 16.42	<0.001
Gender-female, *n* (%)	13348 (51.6)	3746 (72.3)	3269 (63.1)	2785 (53.8)	2274 (43.9)	1274 (24.6)	<0.001
Race, *n* (%)							<0.001
Mexican American	3718 (14.4)	684 (13.2)	652 (12.6)	732 (14.1)	768 (14.8)	882 (17.0)	
Other Hispanic	2643 (10.2)	606 (11.7)	560 (10.8)	510 (9.9)	487 (9.4)	480 (9.3)	
Non-Hispanic White	11162 (43.1)	2071 (40.0)	2274 (43.9)	2284 (44.1)	2324 (44.9)	2209 (42.7)	
Non-Hispanic Black	5587 (21.6)	1293 (25.0)	1158 (22.4)	1101 (21.3)	1006 (19.4)	1029 (19.9)	
Other race including multiracial	2780 (10.7)	525 (10.1)	535 (10.3)	550 (10.6)	592 (11.4)	578 (11.2)	
Body mass index, kg/m^2^	29.39 ± 7.00	29.53 ± 7.01	29.34 ± 7.08	29.50 ± 7.16	29.33 ± 6.92	29.25 ± 6.81	0.202
Ratio of family income to poverty	2.54 ± 1.63	2.21 ± 1.56	2.49 ± 1.60	2.61 ± 1.63	2.71 ± 1.64	2.65 ± 1.65	<0.001
Systolic blood pressure, mmHg	124.40 ± 18.55	124.95 ± 20.41	124.47 ± 19.32	124.40 ± 18.42	124.12 ± 17.70	124.08 ± 16.65	0.124
Diastolic blood pressure, mmHg	70.31 ± 12.74	69.51 ± 13.21	69.54 ± 12.98	70.12 ± 12.53	70.80 ± 12.39	71.58 ± 12.46	<0.001
Total choline, mg	325.44 ± 160.57	148.48 ± 36.09	230.41 ± 18.94	296.23 ± 20.07	379.10 ± 29.00	573.03 ± 143.60	<0.001
Married, *n* (%)	13423 (51.8)	2353 (45.4)	2624 (50.7)	2738 (52.9)	2913 (56.3)	2795 (54.0)	<0.001
Education (high school or above), *n* (%)	20017 (77.3)	3743 (72.3)	3958 (76.4)	4089 (79.0)	4149 (80.1)	4078 (78.8)	<0.001
High blood cholesterol level, *n* (%)	9101 (35.2)	1797 (34.7)	1899 (36.7)	1904 (36.8)	1822 (35.2)	1679 (32.4)	<0.001
Diabetes, *n* (%)	3517 (13.6)	780 (15.1)	709 (13.7)	706 (13.6)	669 (12.9)	653 (12.6)	0.003
eGFR, mg/min/1.73m^2^	93.08 ± 23.84	92.49 ± 25.96	92.07 ± 24.77	92.61 ± 23.49	93.15 ± 23.12	95.06 ± 21.55	<0.001
Moderate work activity, *n* (%)	9641 (37.2)	1652 (31.9)	1771 (34.2)	1941 (37.5)	2010 (38.8)	2267 (43.8)	<0.001
Smoking, *n* (%)	11505 (44.4)	2138 (41.3)	2167 (41.8)	2226 (43.0)	2376 (45.9)	2598 (50.2)	<0.001
Antihypertensive drugs, *n* (%)	8594 (33.2)	1825 (35.2)	1886 (36.4)	1712 (33.1)	1641 (31.7)	1530 (29.5)	<0.001
Total calories, kcal	2016.37 ± 821.40	1296.52 ± 436.42	1707.43 ± 482.85	1960.55 ± 540.66	2249.64 ± 630.09	2867.95 ± 929.73	<0.001
Protein, gm	79.35 ± 34.85	44.36 ± 13.56	63.24 ± 14.33	76.59 ± 17.73	90.28 ± 21.40	122.31 ± 38.14	<0.001
Fat, gm	76.91 ± 38.01	45.98 ± 20.17	63.52 ± 23.67	74.20 ± 26.68	87.24 ± 30.96	113.64 ± 44.60	<0.001
Na, mg	3345.91 ± 1480.89	2096.03 ± 770.91	2786.99 ± 873.28	3270.76 ± 1045.83	3738.79 ± 1177.78	4837.42 ± 1693.79	<0.001
Cholesterol, mg	288.05 ± 189.93	115.14 ± 51.36	187.48 ± 64.57	252.65 ± 80.06	340.17 ± 103.47	544.85 ± 215.66	<0.001
Folate, mcg	176.75 ± 152.00	130.16 ± 107.72	164.06 ± 130.25	182.66 ± 147.09	192.95 ± 166.32	213.94 ± 183.46	<0.001
Vitamins B6, mg	2.04 ± 1.30	1.25 ± 0.94	1.69 ± 0.92	2.00 ± 1.09	2.28 ± 1.18	2.99 ± 1.55	<0.001
Vitamins B12, mg	4.90 ± 4.71	2.59 ± 2.27	3.77 ± 2.55	4.64 ± 3.17	5.55 ± 3.95	7.94 ± 7.58	<0.001
Hypertension, *n* (%)	11032 (42.6)	2310 (44.6)	2341 (45.2)	2188 (42.3)	2154 (41.6)	2039 (39.4)	<0.001

Abbreviations: *n*: number. Values are mean ± standardized differences or *n* (%). a: continuous variables and categorical variables were tested by linear and logistic regression, respectively.

**Table 2:** Logistic regression of total choline intake with odds of hypertension.

Total choline intake, mg	Model I			Model II		Model III			
	OR	95% CI	*p* value	OR	95% CI	*p* value	OR	95% CI	*p* value
As continuous variables (per 100 mg increment)	0.95	(0.94, 0.97)	<0.001	1.00	(0.98, 1.02)	0.826	1.00	(0.95, 1.06)	0.87
As categorical variables (quintiles)									
Q1 (0.15-196.9) (reference)	1.00			1.00			1.00		
Q2 (196.9-262.95)	1.02	(0.95, 1.11)	0.54	1.01	(0.93, 1.11)	0.753	1.04	(0.94, 1.15)	0.410
Q3 (263.0-332.8)	0.91	(0.84, 0.98)	0.016	0.90	(0.82, 0.99)	0.033	0.91	(0.81, 1.01)	0.080
Q4 (332.85-434.25)	0.88	(0.82, 0.96)	0.002	0.93	(0.85, 1.03)	0.156	0.94	(0.83, 1.07)	0.357
Q5 (434.3-2211.75)	0.81	(0.75, 0.87)	<0.001	0.98	(0.89, 1.08)	0.627	0.93	(0.79, 1.11)	0.447
^a^ *p* for trend	<0.001			0.229			0.143		

Model I adjusts for none. Model II adjusts for age, gender, and race. Model III adjusts for age, gender, race, body mass index, ratio of family income to poverty, married status, education, high blood cholesterol level, diabetes, eGFR, moderate work activity, smoking, total calories, protein, fat, Na, cholesterol, folate, vitamin B6, and vitamins B12. a: tests for linear trends were performed by entering the mean value of each quintile group of TC as a continuous variable.

**Table 3:** Linear regression of total choline with systolic blood pressure after excluding patients using antihypertensive drug (*n* = 17296).

Total choline intake, mg	Model I			Model II			Model III		
	*β*	SE	*p* value	*β*	SE	*p* value	*β*	SE	*p* value
As continuous variables (per 100 mg increment)	0.321	0.072	<0.001	-0.018	0.069	0.794	0.064	0.181	0.722
As categorical variables (quintiles)									
Q1 (0.15-196.9) (reference)	0.00			0.00			0.00		
Q2 (196.9-262.95)	-0.252	0.385	0.513	-0.746	0.344	0.030	-0.593	0.353	0.093
Q3 (263.0-332.8)	0.307	0.38	0.419	-0.811	0.342	0.018	-0.816	0.379	0.031
Q4 (332.85-434.25)	0.54	0.378	0.154	-0.998	0.344	0.004	-1.084	0.429	0.012
Q5 (434.3-2211.75)	1.072	0.375	0.004	-0.721	0.353	0.041	-1.308	0.59	0.027
^a^ *p* for trend	<0.001			0.034			0.014		

Model I adjusts for none. Model II adjusts for age, gender, and race. Model III adjusts for age, gender, race, body mass index, ratio of family income to poverty, married status, education, high blood cholesterol level, diabetes, eGFR, moderate work activity, smoking, total calories, protein, fat, Na, cholesterol, folate, vitamin B6, and vitamin B12. a: tests for linear trends were performed by entering the mean value of each quintile group of TC as a continuous variable.

**Table 4:** Linear regression of total choline with DBP after excluding patients using antihypertensive drug (*n* = 17296).

Total choline intake, mg	Model I			Model II			Model III		
	*β*	SE	*p* value	*β*	SE	*p* value	*β*	SE	*p* value
As continuous variables (per 100 mg increment)	0.392	0.053	<0.001	0.158	0.056	0.005	0.277	0.156	0.075
As categorical variables (quintiles)									
Q1 (0.15-196.9) (reference)	0.00			0.00			0.00		
Q2 (196.9-262.95)	-0.034	0.281	0.903	-0.276	0.28	0.324	-0.338	0.308	0.273
Q3 (263.0-332.8)	0.614	0.278	0.027	0.15	0.278	0.590	0.034	0.33	0.917
Q4 (332.85-434.25)	1.281	0.276	<0.001	0.575	0.28	0.040	0.415	0.372	0.264
Q5 (434.3-2211.75)	1.74	0.274	<0.001	0.62	0.287	0.031	0.588	0.511	0.250
^a^ *p* for trend	<0.001			0.001			0.102		

Model I adjusts for none. Model II adjusts for age, gender, and race. Model III adjusts for age, gender, race, body mass index, ratio of family income to poverty, married status, education, high blood cholesterol level, diabetes, eGFR, moderate work activity, smoking, total calories, protein, fat, Na, cholesterol, folate, vitamin B6, and vitamin B12. a: tests for linear trends were performed by entering the mean value of each quintile group of TC as a continuous variable.

**Table 5:** Linear regression of total choline with systolic blood pressure after excluding patients using antihypertensive drug by subgroups.

	Number	Total choline intake, mg									^a^ *p* for interaction
		Q1	Q2		Q3		Q4		Q5		
			*β* (SE)	*p* value	*β* (SE)	*p* value	*β* (SE)	*p* value	*β* (SE)	*p* value	
Gender											0.051
Male	8534	Ref	-0.506 (0.641)	0.43	0.275 (0.639)	0.667	-0.457 (0.665)	0.492	-0.442 (0.814)	0.587	
Female	8762	Ref	-0.292 (0.487)	0.548	-1.031 (0.581)	0.076	-0.97 (0.736)	0.188	-2.035 (1.091)	0.062	
Age, years											<0.001
≥60	3703	Ref	-0.303 (1.066)	0.776	-0.308 (1.187)	0.796	-0.216 (1.406)	0.878	-2.444 (2.026)	0.228	
< 60	13593	Ref	0.115 (0.384)	0.764	0.503 (0.407)	0.217	0.385 (0.456)	0.398	1.176 (0.618)	0.057	
Smoking											0.340
No	10091	Ref	-0.446 (0.467)	0.34	-0.785 (0.509)	0.123	-1.454 (0.589)	0.014	-1.494 (0.827)	0.071	
Yes	7205	Ref	-0.27 (0.618)	0.662	-0.339 (0.647)	0.6	-0.362 (0.711)	0.61	-1.033 (0.948)	0.276	
BMI, kg/m^2^											0.015
≥25	5729	Ref	-0.638 (0.628)	0.309	-0.31 (0.677)	0.647	-0.504 (0.765)	0.51	-1.4 (1.064)	0.188	
<25	11468	Ref	-0.421 (0.47)	0.37	-0.952 (0.501)	0.057	-1.474 (0.566)	0.009	-1.729 (0.771)	0.025	
eGFR, mg/min/1.73 m^2^											<0.001
≥90	11469	Ref	-0.218 (0.406)	0.592	-0.424 (0.435)	0.33	-0.538 (0.489)	0.271	-0.742 (0.666)	0.266	
< 90	5059	Ref	-0.677 (0.797)	0.396	-0.539 (0.852)	0.527	-1.398 (0.977)	0.153	-1.792 (1.363)	0.189	

Data are presented as beta (SE) and *p* value. When analyzing a subgroup variable, age, gender, race, body mass index, ratio of family income to poverty, married status, education, high blood cholesterol level, diabetes, eGFR, moderate work activity, smoking, total calories, protein, fat, Na, cholesterol, folate, vitamin B6, and vitamin B12 were adjusted except for the variable itself. a: *p* for interaction was calculated by using the Wald test statistic.

## Data Availability

The publicly available data analyzed in this study can be found at https://www.cdc.gov/nchs/nhanes/index.htm.

## Supplementary Materials

Supplementary Materials Figure S1: scatter plots of systolic pressure (a) and diastolic pressure (b) versus choline intake. Supplementary Table S1: linear regression of total choline with systolic blood pressure without excluding patients using antihypertensive drug (*n* = 25890).

## References

[1] Rapsomaniki E., Timmis A., George J. (2014). Blood pressure and incidence of twelve cardiovascular diseases: lifetime risks, healthy life-years lost, and age-specific associations in 1∗25 million people. *Lancet*.

[2] Sever P. S., Dahlöf B., Poulter N. R. (2003). Prevention of coronary and stroke events with atorvastatin in hypertensive patients who have average or lower-than-average cholesterol concentrations, in the Anglo-Scandinavian Cardiac Outcomes Trial--Lipid Lowering Arm (ASCOT-LLA): a multicentre randomised controlled trial. *Lancet*.

[3] Gibbons G. H., Dzau V. J. (1994). The emerging concept of vascular remodeling. *The New England Journal of Medicine*.

[4] Brandes R. P. (2014). Endothelial dysfunction and hypertension. *Hypertension*.

[5] Zeisel S. H., da Costa K. A. (2009). Choline: an essential nutrient for public health. *Nutrition Reviews*.

[6] Zeisel S. H., Mar M. H., Howe J. C., Holden J. M. (2003). Concentrations of choline-containing compounds and betaine in common foods. *The Journal of Nutrition*.

[7] Zeisel S. H., Warrier M. (2017). Trimethylamine N-Oxide, the microbiome, and heart and kidney disease. *Annual Review of Nutrition*.

[8] Wang Z., Klipfell E., Bennett B. J. (2011). Gut flora metabolism of phosphatidylcholine promotes cardiovascular disease. *Nature*.

[9] Wang Z., Tang W. H., Buffa J. A. (2014). Prognostic value of choline and betaine depends on intestinal microbiota-generated metabolite trimethylamine-N-oxide. *European Heart Journal*.

[10] Tang W. H., Wang Z., Levison B. S. (2013). Intestinal microbial metabolism of phosphatidylcholine and cardiovascular risk. *The New England Journal of Medicine*.

[11] Meyer K. A., Shea J. W. (2017). Dietary choline and betaine and risk of CVD: a systematic review and meta-analysis of prospective studies. *Nutrients*.

[12] Zheng Y., Li Y., Rimm E. B. (2016). Dietary phosphatidylcholine and risk of all-cause and cardiovascular-specific mortality among US women and men. *The American Journal of Clinical Nutrition*.

[13] Nagata C., Wada K., Tamura T. (2015). Choline and betaine intakes are not associated with cardiovascular disease mortality risk in Japanese men and women. *The Journal of Nutrition*.

[14] Ye J. Z., Li Y. T., Wu W. R. (2018). Dynamic alterations in the gut microbiota and metabolome during the development of methionine-choline-deficient diet-induced nonalcoholic steatohepatitis. *World Journal of Gastroenterology*.

[15] Younossi Z. M., Koenig A. B., Abdelatif D., Fazel Y., Henry L., Wymer M. (2016). Global epidemiology of nonalcoholic fatty liver disease—Meta-analytic assessment of prevalence, incidence, and outcomes. *Hepatology*.

[16] Schneider K. M., Mohs A., Kilic K. (2019). Intestinal microbiota protects against MCD diet-induced steatohepatitis. *International Journal of Molecular Sciences*.

[17] Ufnal M., Jazwiec R., Dadlez M., Drapala A., Sikora M., Skrzypecki J. (2014). Trimethylamine-N-oxide: a carnitine-derived metabolite that prolongs the hypertensive effect of angiotensin II in rats. *The Canadian Journal of Cardiology*.

[18] Jiang S., Shui Y., Cui Y. (2021). Gut microbiota dependent trimethylamine N-oxide aggravates angiotensin II- induced hypertension. *Redox Biology*.

[19] Taesuwan S., Vermeylen F., Caudill M. A., Cassano P. A. (2019). Relation of choline intake with blood pressure in the National Health and Nutrition Examination Survey 2007-2010. *The American Journal of Clinical Nutrition*.

[20] Golzarand M., Bahadoran Z., Mirmiran P., Azizi F. (2021). Dietary choline and betaine intake and risk of hypertension development: a 7.4-year follow-up. *Food & Function*.

[21] Taesuwan S., Thammapichai P., Ganz A. B., Jirarattanarangsri W., Khemacheewakul J., Leksawasdi N. (2022). Associations of choline intake with hypertension and blood pressure among older adults in cross-sectional 2011-2014 National Health and Nutrition Examination Survey (NHANES) differ by BMI and comorbidity status. *The British Journal of Nutrition*.

[22] Zipf G., Chiappa M., Porter K. S., Ostchega Y., Lewis B. G., Dostal J. (2013). *National health and nutrition examination survey: plan and operations, 1999-2010*.

[23] Ahluwalia N., Dwyer J., Terry A., Moshfegh A., Johnson C. (2016). Update on NHANES dietary data: focus on collection, release, analytical considerations, and uses to inform public policy. *Advances in Nutrition*.

[24] Stevens L. A., Claybon M. A., Schmid C. H. (2011). Evaluation of the Chronic Kidney Disease Epidemiology Collaboration equation for estimating the glomerular filtration rate in multiple ethnicities. *Kidney International*.

[25] Gawrys-Kopczynska M., Konop M., Maksymiuk K. (2020). TMAO, a seafood-derived molecule, produces diuresis and reduces mortality in heart failure rats. *eLife*.

[26] Huc T., Drapala A., Gawrys M. (2018). Chronic, low-dose TMAO treatment reduces diastolic dysfunction and heart fibrosis in hypertensive rats. *American Journal of Physiology. Heart and Circulatory Physiology*.

[27] Zeisel S. H. (1990). Choline deficiency. *The Journal of Nutritional Biochemistry*.

[28] Zeisel S. H., daCosta K. A., Youssef M., Hensey S. (1989). Conversion of dietary choline to trimethylamine and dimethylamine in rats: dose-response relationship. *The Journal of Nutrition*.

[29] Zeisel S. H., Wishnok J. S., Blusztajn J. K. (1983). Formation of methylamines from ingested choline and lecithin. *The Journal of Pharmacology and Experimental Therapeutics*.

[30] Rath S., Heidrich B., Pieper D. H., Vital M. (2017). Uncovering the trimethylamine-producing bacteria of the human gut microbiota. *Microbiome*.

[31] Romano K. A., Vivas E. I., Amador-Noguez D., Rey F. E. (2015). Intestinal microbiota composition modulates choline bioavailability from diet and accumulation of the proatherogenic metabolite trimethylamine-*N*-oxide. *MBio*.

[32] Shang Y., Wang X., Shang X. (2016). Repetitive transcranial magnetic stimulation effectively facilitates spatial cognition and synaptic plasticity associated with increasing the levels of BDNF and synaptic proteins in Wistar rats. *Neurobiology of Learning and Memory*.

[33] Cho C. E., Taesuwan S., Malysheva O. V. (2017). Back cover: Trimethylamine-N-oxide (TMAO) response to animal source foods varies among healthy young men and is influenced by their gut microbiota composition: a randomized controlled trial. *Molecular Nutrition & Food Research*.

[34] Tyakht A. V., Kostryukova E. S., Popenko A. S. (2013). Human gut microbiota community structures in urban and rural populations in Russia. *Nature Communications*.

[35] Millard H. R., Musani S. K., Dibaba D. T. (2018). Dietary choline and betaine; associations with subclinical markers of cardiovascular disease risk and incidence of CVD, coronary heart disease and stroke: the Jackson Heart Study. *European Journal of Nutrition*.

[36] Detopoulou P., Panagiotakos D. B., Antonopoulou S., Pitsavos C., Stefanadis C. (2008). Dietary choline and betaine intakes in relation to concentrations of inflammatory markers in healthy adults: the ATTICA study. *The American Journal of Clinical Nutrition*.

[37] Pan X. F., Yang J. J., Shu X. O. (2021). Associations of circulating choline and its related metabolites with cardiometabolic biomarkers: an international pooled analysis. *The American Journal of Clinical Nutrition*.

[38] Ali M. A., Nasir M., Pasha T. N. (2020). Association of life style and dietary habits with blood choline and cardiovascular outcome. *Cellular and Molecular Biology (Noisy-le-Grand, France)*.

[39] Van Parys A., Lysne V., Svingen G. F. T. (2020). Dietary choline is related to increased risk of acute myocardial infarction in patients with stable angina pectoris. *Biochimie*.

[40] Mazidi M., Katsiki N., Mikhailidis D. P., Banach M. (2019). Dietary choline is positively related to overall and cause-specific mortality: results from individuals of the National Health and Nutrition Examination Survey and pooling prospective data. *The British Journal of Nutrition*.

[41] Yang J. J., Lipworth L. P., Shu X. O. (2020). Associations of choline-related nutrients with cardiometabolic and all-cause mortality: results from 3 prospective cohort studies of blacks, whites, and Chinese. *The American Journal of Clinical Nutrition*.

[42] Heianza Y., Ma W., Manson J. E., Rexrode K. M., Qi L. (2017). Gut microbiota metabolites and risk of major adverse cardiovascular disease events and death: a systematic review and meta-analysis of prospective studies. *Journal of the American Heart Association*.

[43] Bennett B. J., de Aguiar Vallim T. Q., Wang Z. (2013). Trimethylamine-N-oxide, a metabolite associated with atherosclerosis, exhibits complex genetic and dietary regulation. *Cell Metabolism*.

[44] Farhangi M. A., Vajdi M. (2020). Novel findings of the association between gut microbiota-derived metabolite trimethylamine N-oxide and inflammation: results from a systematic review and dose-response meta-analysis. *Critical Reviews in Food Science and Nutrition*.

[45] Papandreou C., Moré M., Bellamine A. (2020). Trimethylamine N-oxide in relation to cardiometabolic health-cause or effect?. *Nutrients*.

[46] Tang W. H., Hazen S. L. (2014). The contributory role of gut microbiota in cardiovascular disease. *The Journal of Clinical Investigation*.

[47] Zhuang R., Ge X., Han L. (2019). Gut microbe–generated metabolite trimethylamine N-oxide and the risk of diabetes: a systematic review and dose-response meta-analysis. *Obesity Reviews*.

[48] Chhibber-Goel J., Singhal V., Parakh N., Bhargava B., Sharma A. (2017). The metabolite trimethylamine-N-oxide is an emergent biomarker of human health. *Current Medicinal Chemistry*.

[49] Janeiro M. H., Ramírez M. J., Milagro F. I., Martínez J. A., Solas M. (2018). Implication of trimethylamine N-oxide (TMAO) in disease: potential biomarker or new therapeutic target. *Nutrients*.

[50] Ma J., Pazos I. M., Gai F. (2014). Microscopic insights into the protein-stabilizing effect of trimethylamine N-oxide (TMAO). *Proceedings of the National Academy of Sciences of the United States of America*.

[51] Dumas M. E., Rothwell A. R., Hoyles L. (2017). Microbial-host co-metabolites are prodromal markers predicting phenotypic heterogeneity in behavior, obesity, and impaired glucose tolerance. *Cell Reports*.

