# Supplementary material for: Association of Choline Intake with Blood Pressure and Effects of Its Microbiota-Dependent Metabolite Trimethylamine-N-Oxide on Hypertension
Source: Cardiovasc Ther. 2022 Aug 25;2022:9512401. doi: 10.1155/2022/9512401 (PMC9436605; doi:10.1155/2022/9512401)
Supplement: Supplementary Materials — Figure S1: scatter plots of systolic pressure (a) and diastolic pressure (b) versus choline intake. Supplementary Table S1: linear regression of total choline with systolic blood pressure without excluding patients using antihypertensive drug (n = 25890). [file 9512401.f1.zip › Supplementary Table S1.pdf]

Supplementary Table S1 Linear regression of total choline with systolic blood pressure without excluding patients using antihypertensive drug (n=25890)

Total choline intake, mg	Model I			Model II			Model III		
	β	SE	p-value	β	SE	p-value	β	SE	p-value
As continuous variables (per 100mg increment)	-0.145	0.072	0.043	-0.06	0.068	0.376	-0.274	0.177	0.121
As categorical variables (quintiles)									
Q1 (0.15-196.9) (Reference)	0.00			0.00			0.00		
Q2(196.9-262.95)	-0.481	0.364	0.187	-0.826	0.324	0.011	-0.927	0.332	0.005
Q3 (263.0-332.8)	-0.55	0.364	0.131	-0.938	0.326	0.004	-1.011	0.363	0.005
Q4 (332.85-434.25)	-0.823	0.364	0.024	-1.104	0.33	0.001	-1.297	0.416	0.002
Q5 (434.3-2211.75)	-0.865	0.364	0.018	-0.769	0.34	0.024	-1.618	0.576	0.005
P for trend	0.011			0.016			0.004		

Model I adjust for none. Model II adjust for Age, Gender, and Race. Model III adjust for Age, Gender, Race, Body mass index, Ratio of family income to poverty, Married status, Education, High blood cholesterol level, Diabetes, eGFR, Moderate work activity, smoking, Total calories, Protein, Fat, Na, Cholesterol, Folate, Vitamins B6, and Vitamins B12, Antihypertensive drugs). a: Tests for linear trends were performed by entering the mean value of each quintile group of TC as a continuous variable.
