# Supplementary figures and images for: Association of Choline Intake with Blood Pressure and Effects of Its Microbiota-Dependent Metabolite Trimethylamine-N-Oxide on Hypertension
Source: Cardiovasc Ther. 2022 Aug 25;2022:9512401. doi: 10.1155/2022/9512401 (PMC9436605; doi:10.1155/2022/9512401)

**a**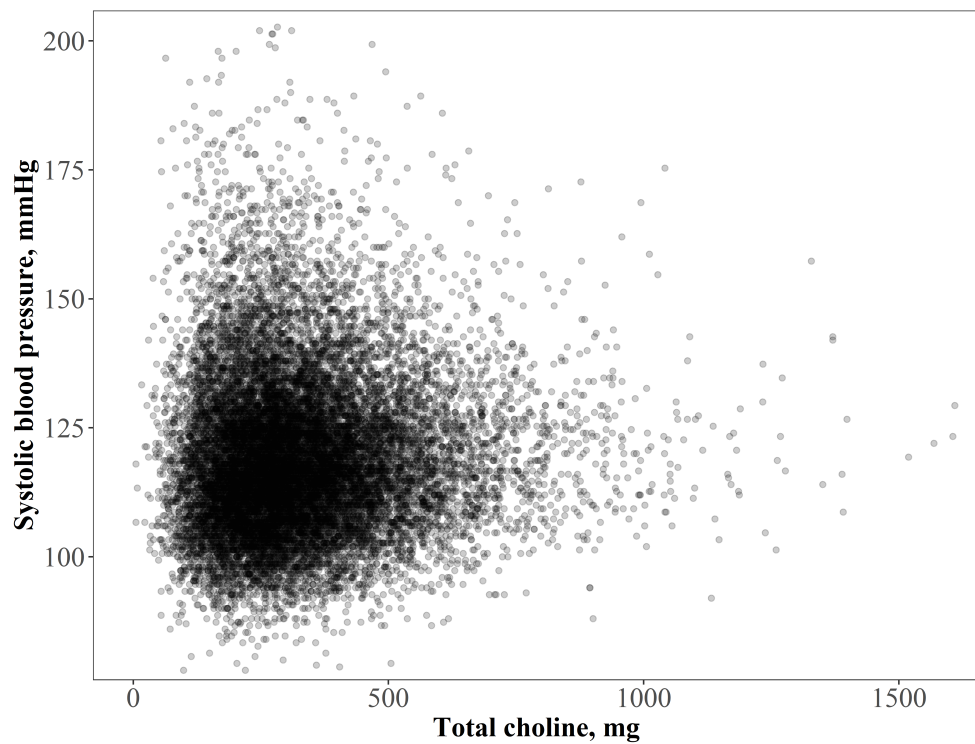**b**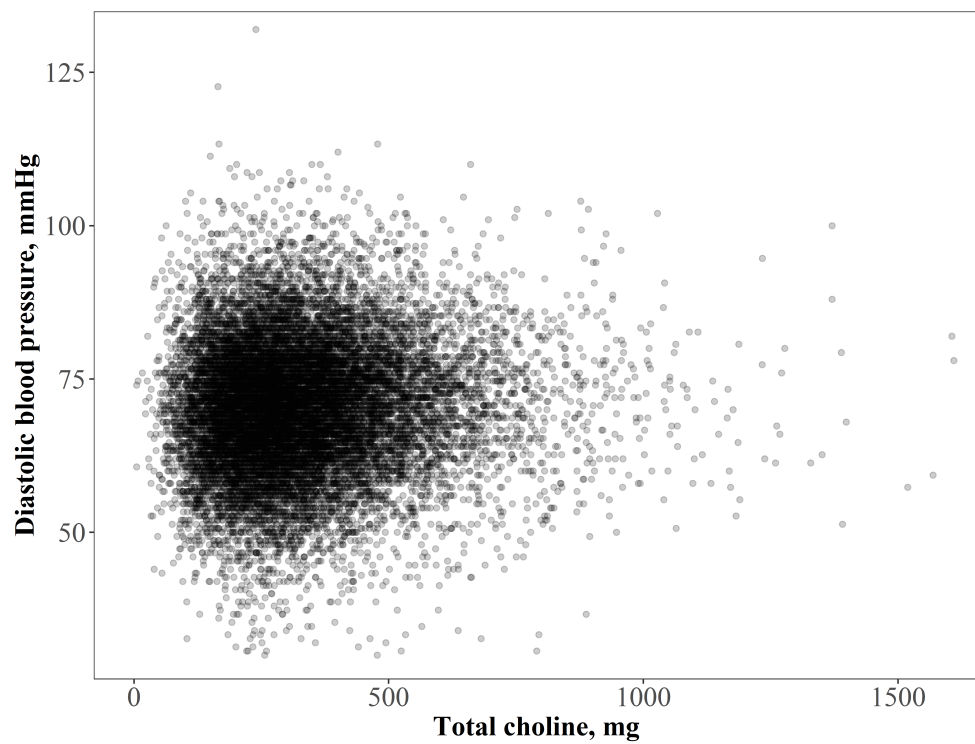

Supplement: Supplementary Materials — Figure S1: scatter plots of systolic pressure (a) and diastolic pressure (b) versus choline intake. Supplementary Table S1: linear regression of total choline with systolic blood pressure without excluding patients using antihypertensive drug (n = 25890). [file 9512401.f1.zip › Supplementary Figure S1.pdf]
